# Primary Hepatic Lymphoma

**DOI:** 10.1007/s12029-013-9505-7

**Published:** 2013-05-18

**Authors:** Aziz Zentar, Mohamed Tarchouli, Hakim Elkaoui, Mohamed Said Belhamidi, Moulay Brahim Ratbi, Sidi Mohamed Bouchentouf, Abdelmounaim Ait Ali, Ahmed Bounaim, Khalid Sair

**Affiliations:** Department of Digestive Surgery I, Mohammed V Military Hospital, Mohammed V—Souissi University, Rabat, Morocco

## Introduction

Primary hepatic lymphoma (PHL) is confined to the liver with no evidence of lymphomatous involvement in other lymphoid structures. It is a very rare malignancy representing less than 1 % of all extra nodal lymphomas. The exact cause of PHL is unknown, but it seems that there is a strong association between hepatitis C virus (HCV) and PHL. The majority of PHL patients are middle-aged men who usually present nonspecific symptoms. Diagnosis of PHL requires a liver biopsy compatible with lymphoma and the absence of lympho-proliferative disease outside the liver. The rarity of the disease leads to problems of diagnosis and management. The optimal treatment is still unclear and the results are uncertain.

We report a case of PHL developed in segment I of the liver, and we present a review of the literature including clinical, radiological, histological, and therapeutic features of this disease.

This case report is approved by the patient herself. Informed consent has been obtained from the patient for publication of this case.

## Case Report

A 65-year-old woman, without medical history, presented, 4 months earlier with abdominal pain associated with a long-term fever and weight loss. The patient had no complaints of vomiting, jaundice, or night sweats.

The physical examination on admission found an asthenic patient with mild mucocutaneous pallor, a temperature of 38.5 °C and blood pressure of 120/70 mmHg. There was also a slight abdominal distension but no signs of cirrhosis or portal hypertension. The liver and spleen were normal in size and lymph nodes were not enlarged.

Laboratory results including a blood cell count, an assessment of cytolysis and cholestasis did not reveal any abnormalities. However, serology was positive for hepatitis C with a slightly elevated C-reactive protein levels at 60 mg/l. Tumor markers (alpha-fetoprotein and carcinoembryonic antigen) were normal. The chest X-ray was unremarkable.

**Fig. 1:**
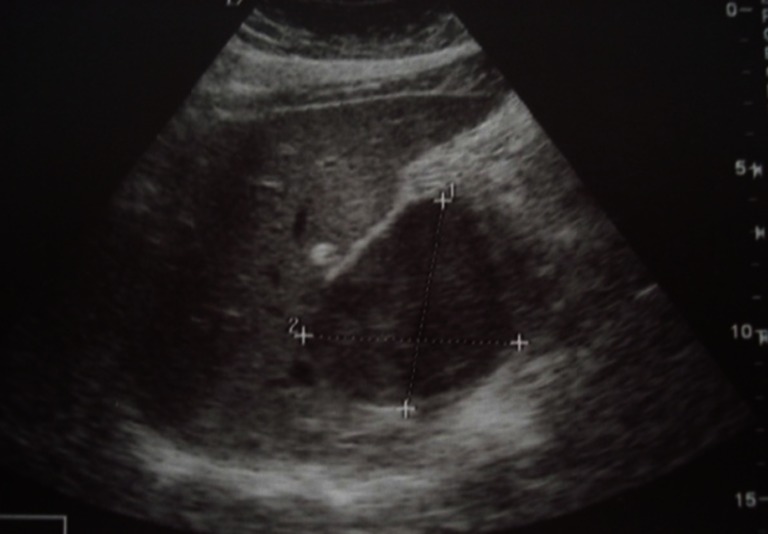
Abdominal ultrasound image showing a hypo-echoic mass developed in segment I of the liver, with regular outlines

**Fig. 2:**
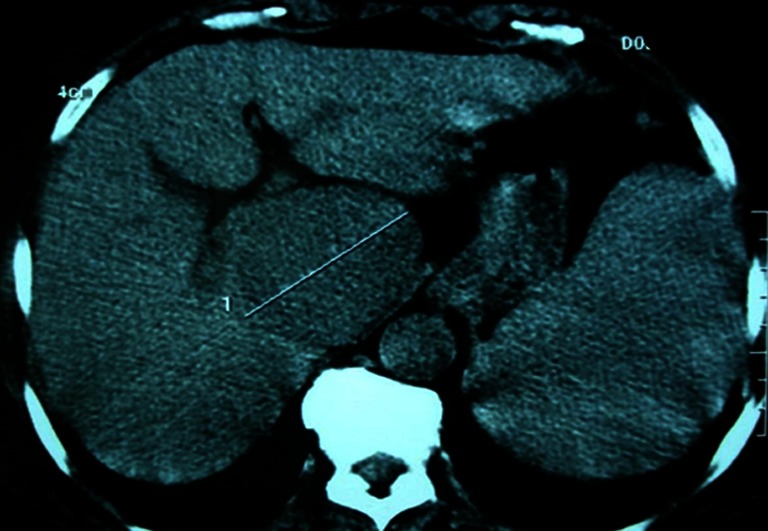
Abdominal CT scan without contrast showing a hypo-dense mass with regular contours, measuring 10 × 7 cm at the expense of the caudate lobe of the liver

**Fig. 3:**
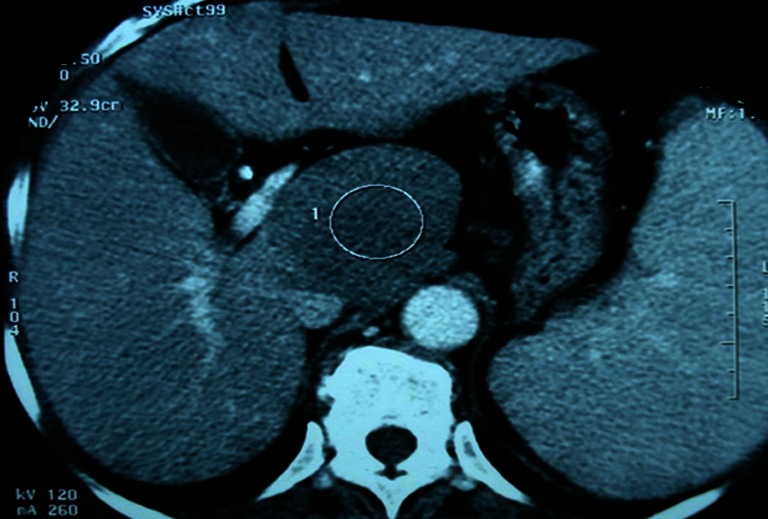
Post-contrast abdominal CT scan shows the lesion with a well-demarcated margin taking just the contrast with peripheral vascular enhancement

Abdominal ultrasound showed a hypo-echoic tissue mass developed in the caudate hepatic lobe with a well-demarcated margin (Fig. 1). The abdominal computed tomography (CT) scan revealed that the tumor was hypo-dense, well limited in the pre-contrast study and taking just the contrast with peripheral vascular enhancement evoking a hepatocellular carcinoma in the post-contrast study (Figs. 2 and 3). There was no evidence of biliary or pancreatic disease, splenomegaly, or abdominal lymphadenopathy. Radiography and CT scan of the chest did not reveal any mediastinal lymphadenopathy. Endoscopic examinations of the upper and lower gastrointestinal tracts were performed but no abnormality was found.

Surgical exploration was indicated for hepatic carcinoma evolving on an apparently healthy liver with a negative staging. During the intervention, it was found that there was an encapsulated liver tumor at the expense of segment I. The extra-tumor liver was healthy. Hepatic segmentectomy was performed. Macroscopic study of the piece showed a hepatic mass well-limited encapsulated with soft consistency measuring 11.5 × 8 × 7 cm. The non-tumor part of the liver parenchyma was normal. Microscopically, there was a heavy infiltration composed mainly of large lymphoid cells. Immunohistochemical staining demonstrated that the cells were positive for CD20 and leukocyte common antigen. Subsequently, a bone marrow biopsy was performed. It showed normal cellularity with normal maturation of all cell lines. Thus, according to the absence of other foci of lymphoma, a diagnosis of diffuse large B cell primary hepatic lymphoma was established. The patient was referred to oncology for chemotherapy. She received 8 cycles of CHOP protocol (cyclophosphamide–doxorubicin–vincristine–prednisolone). The postoperative course is simple. The patient is currently in 2-years follow-up with a good evaluation.

## Discussion

Primary hepatic lymphoma (PHL) is defined as an extranodal lymphoma of the liver without involvement of any other organ (lymph node, bone marrow, spleen, etc.) [1, 2]. The primary non-Hodgkin lymphoma of the liver is a rare condition that represents less than 1 % of all extra nodal lymphomas [1]. It affects preferentially men (sex ratio of three men to one woman) of mean age 50 years [3].

The etiology of this disease is still unknown. However, the presence of chronic inflammation of the liver parenchyma (hepatitis B or C), an EBV infection, or immunosuppression (human immunodeficiency virus HIV) seems to play an important role in the pathogenesis of PHL [4–6]. Hepatitis C is found in 40–60 % of patients with PHL [1], suggesting that there is a strong association between HCV and non-Hodgkin lymphoma of the liver. Our patient had hepatitis C, thus this virus may play a role in the genesis of the disease.

Presentations vary from the incidental discovery in otherwise asymptomatic patients to onset of fulminant hepatic failure with rapid progression of encephalopathy to coma and death. The symptoms are usually nonspecific. Patients may report abdominal pain (right upper quadrant or epigastric pain), nausea, fever, asthenia, weight loss, and anorexia. Hepatomegaly is often present on clinical examination but jaundice is rarely found.

Based on liver infiltration, PHL can be subdivided into nodular or diffuse types. The predominant histology of PHL is diffuse large B cell lymphoma [7, 8], as was the case for our patient. Other histologic types described (less than 5 % of cases) are immunoblastic, lymphoblastic, Burkett’s lymphomas, mucosa-associated lymphoid tissue lymphomas, anaplastic large-cell lymphoma, and rare cases of lymphoma T cell [8].

Patients with PHL typically have abnormal liver function tests, with elevation of lactate dehydrogenase (LDH) and alkaline phosphatase. Hypercalcemia is observed in 40 % of patients for unknown reasons, but the release of calcitriol by malignant lymphoma cells is considered as a possible cause [1, 2, 9–11]. In the case of hepatic tumor, elevated LDH, with normal alpha-fetoprotein (AFP) and carcinoembryonic antigen (CEA), remains a valuable biologic feature [5, 7]. In our case, there are no biological abnormalities apart from a positive serology for hepatitis C.

Ultrasound is a good screening test, showing classically hypo-echoic lesions relative to normal liver [8, 12]. The abdominal CT scan found hypo-attenuating masses, unenhanced or poorly enhanced after contrast. The findings with magnetic resonance imaging are variable, however, as several authors have described lesions that appear hypo-intense on T1-weighted and hyper-intense in T2-weighted [2, 8, 12–14]. Liver biopsy remains the most valuable tool for diagnosis of PHL. If a discrete mass is not visible on imaging for percutaneous liver biopsy, the first transjugular approach may be reasonable. Immunohistochemical typing is needed to differentiate lymphoma from other malignancies. In our patient, liver biopsy was not performed because we did not deem it essential to the near certainty of malignancy made by morphological assessment and inherent risks in this one particular swarming on the puncture tract. With normal level of alpha-fetoprotein, our patient was mistaken as having AFP-negative hepatic cancer before pathological diagnosis. The histological diagnosis of lymphoma on liver biopsy is not always possible; many patients are thus operated for erroneous diagnosis of carcinoma or other tumor. Our observation illustrates this scenario.

Due to the rarity of this disease entity, the nonspecific clinical presentation, and laboratory and radiological features, PHL can be confused with focal nodular hyperplasia, primary hepatic tumors, carcinoma with hepatic metastases, and systemic lymphoma with secondary hepatic involvement [2, 3, 6, 15].

The therapeutic modalities reported in the literature are variable associating surgery, chemotherapy, radiotherapy, or combination of the various processes [2, 3]. However, early surgical treatment followed by chemotherapy seems to give good performances and provide benefits in term of survival [6, 16]. The indications for surgery are not well established, although localized disease can be treated by liver resection [16]. But it requires patients in good general condition in absence of co-morbidities, with preserved liver function and a tumor of small size [3]. Surgery is also used in order to reduce tumor volume before or after chemotherapy [2, 3].

Poor prognostic features include advanced age, constitutional symptoms, bulky disease, unfavorable histologic subtype, elevated levels of LDH and β2-microglobulin, a high proliferation rate, cirrhosis, and comorbid conditions [17]. The appreciation of prognostic factors in our patient through the assessment of his impaired general condition, his advanced age, the absence of concomitant illness, and isolated character of tumor enabled us to predict a moderately good prognosis.

## Conclusion

Primary hepatic lymphoma is a rare entity that should be considered in any patient with liver mass or liver infiltration, especially with normal levels of alpha-fetoprotein and CEA. The rarity of the disease leads to problems of diagnosis and management. The optimal treatment is still unclear and the results are uncertain. Most patients are treated with multi-agent chemotherapy, with some physicians employing a multimodality approach. Surgery may be proposed in the nodular forms, with a postoperative chemotherapy highly recommended to reduce the rate of extra-hepatic recurrence. The prognosis is variable depending on several factors. Suppression from hepatic viral infections could be a curial step in preventing carcinogenesis of PHL.
